# Supplementary material for: Population genetics and demography of the coral-killing cyanobacteriosponge, Terpios hoshinota, in the Indo-West Pacific
Source: PeerJ. 2022 May 31;10:e13451. doi: 10.7717/peerj.13451 (PMC9165603; doi:10.7717/peerj.13451)
Supplement: Supplemental Information 1 [file peerj-10-13451-s001.docx]

**Population genetics and demography of the coral-killing cyanobacteriosponge, *Terpios hoshinota,* in the Indo-West Pacific**

Savanna Wenhua Chow^1,2,3,4*^, Shashank Keshavmurthy^2*^, James Davis Reimer^5,6^, Nicole de Voogd^7,8^, Hui Huang^9^, Jih-Terng Wang^3^, Sen-Lin Tang^2^, Peter J Schupp^10,11^, Chun Hong Tan^12^, Hock-Chark Liew^13^, Keryea Soong^3^, Beginer Subhan^14^, Hawis Madduppa^14^, Chaolun Allen Chen^1,2,4,15^

^1^ Department of Life Sciences, National Taiwan Normal University, Taipei, Taiwan

^2^ Biodiversity Research Center, Academia Sinica, Taipei, Taiwan

^3^ Department of Oceanography, National Sun Yat-Sen University, Kaohsiung, Taiwan

^4^Biodiversity Program, Taiwan International Graduate Program, Academia Sinica, Taipei, Taiwan

^5^Department of Biology, Chemistry, and Marine Science, University of Ryukyus, Naha, Okinawa, Japan

^6^Tropical Biosphere Research Center, , University of the Ryukyus, Okinawa, Japan

^7^Naturalis Biodiversity Center, Leiden, The Netherlands

^8^Institute of Environmental Sciences, Environmental Biology Department, Leiden University, Leiden, Netherlands

^9^CAS Key Laboratory of Tropical Marine Bio-resources and Ecology and Guangdong Provincial Key Laboratory of Applied Marine Biology, South China Sea Institute of Oceanology, Chinese Academy of Sciences, Guangzhou, China

^10^Institute for Chemistry and Biology of the Marine Environment, University of Oldenburg, Oldenburg, Germany

^11^Helmholtz Institute for Functional Marine Biodiversity at the , University of Oldenburg (HIFMB), 26129, Oldenburg, Germany

^12^School of Marine and Environmental Sceinces, University of Malaysia Terengganu, Terengganu, Malaysia

^13^Sdn Bhd. 326B, Jalan Hiliran, 20300 Kuala Terengganu, Alchemy Laboratory & Services, Terengganu, Malaysia

^14^Department of Marine Science & Technology, Faculty of Fisheries & Marine Sciences, IPB University, 16680, Indonesia, Bogor , Indonesia

^15^Department of Life Science, Tunghai University, Taichung, Taiwan

*These authors contributed equally to this work

Corresponding Authors: Chaolun Allen Chen, Biodiversity Research Centre, Academia Sinica, Nangang, Taipei 115, Taiwan, Tel: 886-2-27899549; E-mail:cac@gate.sinica.edu.tw

**Supplementary Table 1**: Literature review of *T.hoshinota* with major findings and outbreak status inferred from these studies.

Information on the locations around the world from where occurrence and outbreak of *T.hoshinota* has been reported starting from 1973 until present.

| **Country** | **Locality** | **Year** | **Major finding** | **Outbreak status** | **References** |
| --- | --- | --- | --- | --- | --- |
| United States | Guam, Mariana Islands | 1973 | First observation of *Terpios* in Guam | outbreak | Bryan, 1973 |
| Japan | Ryukyu Archipelago | 1984 | First observation of *Terpios* in Bise, Okinawa | outbreak | Rützler et al., 1993 |
|  |  | 1985 | *Terpios* observed in Yonama, Tokunoshima | outbreak | Rützler et al., 1993 |
| United States | Guam, Mariana Islands | 1987 | *Terpios* overgrowth most hard and stable reef substrata, with great potential for covering reefs due to high growth rate with longevity, and toxicity to corals | - | Plucer-Rosario, 1987 |
| Japan | Ryukyu Archipelago | 1993 | Described *Terpios* as “cyanobacteriosponge” with tylostyle spicules. *Terpios* harbors large quantities of symbiont belongs to *Aphanocapsa raspaigellae* type. | - | Plucer-Rosario, 1987 |
| Taiwan | Green Island | 2007 | *Terpios* observed in Chaikou, Green Island | outbreak | Liao et al., 2007 |
| Taiwan | Green Island | 2009 | *Terpios* can utilize pioneering thin tissue threads to overgrowth unsuitable habitats in the shading experiments | - | Soong et al., 2009 |
| Taiwan | Green Island | 2011 | The sponge-associated bacterial community in side *Terpios* was mainly cyanobacteria, and sponge might get benefit from the associated bacterial community with unhealthy coral closest to the margin of the sponge. | - | Reimer et al., 2011 |
| Australia | Lizard Island, Great Barrier Reef | 2011 | The first record of *Terpios* in Great Barrier Reef, suggesting this species may be expanding to south-eastern Pacific. | non-outbreak | Fujii et al., 2011 |
| Japan | Ryukyu Archipelago (Amami Oshima Island to the Yaeyama Islands) | 2011 | The presence of *Terpios* has considerable increase from levels reported before, but large outbreak remains the same as in 1984-1985. | non-outbreak | Reimer et al., 2011 |
| Japan | Yonama, Tokunoshima & Yakomo, Okinoerabu. | 2011 | *Terpios* disappear in the Tokunoshima Island which was found outbreak in 1986, suggest the appearances of this sponge may be irreversible. Newly founded outbreak of *Terpios* in the neighboring island Okinoerabu | outbreak | Reimer et al., 2011 |
| Japan | Shiraho, Ishigakijima Island | 2011 | Spermatic follicles were only found in mesohyl of the sponge in summer, suggesting seasonal reproduction may play crucial role in long-distance dispersal | - | Hirose et al.,2011 |
| China | Yongxing Island, South China Sea | 2012 | The outbreak of *Terpios* in the northern reef caused massive coral mortality | outbreak | Shi et al., 2012 |
| Taiwan | Gon-Guam & Chai-Ko, Green Island | 2012 | The Terpios-coral interaction did not show species specificity, and the overgrowth performance of *Terpios* rely on the viability status of both organisms | - | Wang et al.,  2012 |
| Taiwan | Dabaisha, Green Island | 2012 | Limited swimming capability, rapid settlement and negative buoyancy of *Terpios* larvae suggest the dispersion distance may be limited. | - | Wang et al., 2012 |
| United States | Pagan, Northern Mariana Islands. | 2012 | *Terpios* were observed to expand simultaneously with the cyanobacterial bloom and and increased volcanic activity | outbreak | Schils, 2012 |
| Indonesia | Dapur Island, Java | 2013 | First observation of *Terpios* invasion into Indonesia, overgrowing large patches of corals at the reef of Dapur Island, off Jakarta, Java | non-outbreak | de Voogd et al., 2013 |
| Taiwan | Daibaisha, Green Island | 2013 | *Terpios* larvae may prefer to settle on hard substrate near healthy coral, then juveniles develop to expand and overgrow corals after 7 days. | - | Hsu et al., 2013 |
| Malaysia | Tioman Island | 2014 | *Terpios* prefers to overgrowth coral parts exposed to sunlight. | not mentioned | Hoeksema et al., 2014 |
| Maldives | Faafu Atoll | 2015 | First record of *Terpios* in Maldives and the extension to the Indian Ocean | non-outbreak | Montano et al., 2014 |
| India | Palk Bay | 2015 | first record of *Terpios* invasion in India | outbreak | Thinesh et al., 2015 |
| Taiwan | Gong-guan, Green Island | 2015 | The *Terpios-*cyanobacteria association utilizes not only morphological transformation of  thin tissue threads, but also uses physiological outperformance in accumulating resources | - | Wang et al., 2015 |
| Taiwan | Green Island | 2015 | Draft genome assembly from *Terpios* | - | Yu et al., 2015 |
| Indonesia | Spermonde Archipelago, SW Sulawesi | 2015 | Abundance and genetic variation of the coral-killing cyanobacteriosponge Terpios hoshinota in the Spermonde Archipelago, SW Sulawesi, Indonesia | non-outbreak | van der Ent et al.,2015 |
| Indonesia | Spermonde Archipelago, SW Sulawesi | 2015 | The *Terpios* may have colonization preference on corals of the family Acroporidae and Pocilloporidae. | non-outbreak | van der Ent et al.,2015 |
| Mauritius | Anse La Raie lagoon | 2016 | The most southwestern site for *Terpios* invasion, and the morphological plasticity helps coral species *M. aequituberculata* can overgrowth *Terpios* cololny. | outbreak | Elliott et al., 2015 |
| Mauritius | Anse La Raie lagoon | 2016 | *Terpios* has statistically significant coral substrate preference on branching corals with 5% increment of abundance within 1 month through asexual reproduction. | outbreak | Elliott et al., 2016 |
| Taiwan | Green Island | 2016 | *Terpios* has high fecundity with monthly release larvae during summer. The high fecundity, negative buoyancy and rapid post-settlement growth would result in rapid population expansion | - | Nozawa et al., 2016 |
| Taiwan | Yakomo, Okinoerabu-jima Island | 2017 | Subtropical typhoons may play important roles in the dynamics of outbreak and disappearances. | outbreak | Yomogida et al., 2017 |
| Indonesia | Indonesian archipelago | 2017 | Persistent outbreaks of *Terpios* in Indonesia archipelago coral reefs. | outbreak | Madduppa et al., 2015 |
| India |  | 2017 | *In situ* shading experiments suggest that the symbiotic cyanobacteria acts like nutrient factory for *Terpios* overgrowth on corals. | outbreak | Thinesh et al., 2017 |
| India | Palk Bay, Southeast India | 2017 | *Terpios* has gradual increase in prevalence with high mortality rate to corals. | outbreak | Thinesh et al., 2017 |
| India | Gulf of Mannar, Southeast India | 2018 | Coral reef monitoring started from 2004 shows no *Terpios* invasion until 2018 during this field investigation, and the affected corals mainly belong to montiporids. | outbreak | Raj et al., 2018 |
| Taiwan | Taiping Island (Itu Aba), Spratlys, South China Sea | 2018 | First observation of *Terpios* invasion at the north- western coast of the island | outbreak | Yang et al., 2018 |
| Australia | Berthier Island, West Montalivet Island, Kimberley, Western Australia | 2019 | First Report of the Coral-Killing Sponge *Terpios hoshinota* Rützler and Muzik, 1993 in Western Australia: A New Threat to Kimberley Coral Reefs? | non-outbreak | Fromont et al., 2019 |
| Indonesia | Seribu Islands, Jakarta | 2020 | Growth rate, spatial-temporal variation and prevalence of the encrusting cyanosponge (*Terpios hoshinota*) in Seribu Islands, Jakarta | outbreak | Nugraha et al., 2020 |
| Taiwan | Green Island | 2021 | Testing of how and why the Terpios hoshinota sponge kills stony corals | - | Syue, Hsu & Soong, 2021 |

**Supplementary Table 2**: Sampling number, full name and abbreviation of sampling sites and the collection year of *T.hoshinita*. Sampling sites were grouped based on the geographical region based on marine ecoregion

| **Ecoregion** | **Abbrevations** | **n** | **Sampling site** | **Collection Year** | **References** |
| --- | --- | --- | --- | --- | --- |
| South Kuroshio |  |  |  |  |  |
|  | ARA | 5 | Arahama, Kumejima island, Japan | 2009 | Reimer et al., 2011 |
|  | YAK | 5 | Yakomo, Okinoerabu Island | 2009 | Reimer et al., 2011 |
|  | BSE | 11 | Bise, Okinawa, Japan | 2009 | Reimer et al., 2011 |
|  | SHI | 3 | Shiraho, Ishigaki Island, Japan | 2009 | Reimer et al., 2011 |
|  | MYK | 4 | Miyako, Miyakojima Island, Japan | 2009 | Reimer et al., 2011 |
|  | GI | 41 | Green Island, Taiwan | 2009 | Soong et al., 2009 |
|  | LYU | 35 | Lanyu, Taiwan | 2009 | Soong et al., 2009 |
|  | WLT | 5 | Wanlitong, Kenting, Taiwan | 2014 | Soong et al., 2009 |
|  |  |  |  |  |  |
| South China Sea Ocean Islands | |  |  |  |  |
|  | TP | 6 | Taiping Island (Itu Aba), Spratlys, Taiwan | 2017 | Yang et al., 2018 |
|  | XS | 3 | Xisha (Yongxing), Paracel Islands, China | 2010 | Shi et al., 2012 |
|  |  |  |  |  |  |
| Sunda Shelf |  |  |  |  |  |
|  | MAL | 9 | Terengganu, Malaysia | 2013 | Madduppa et al., 2015 |
|  | NAT | 1 | Natuna, Riau Archipelago, Indonesia | 2017 | Madduppa et al., 2015 |
|  |  |  |  |  |  |
| Southern Java |  |  |  |  |  |
|  | TS | 23 | Thousand islands complex, Java, Indonesia | 2017 | Madduppa et al., 2015 |
|  |  |  |  |  |  |
| Lesser Sunda |  |  |  |  |  |
|  | MBD | 2 | Maluku Barat Daya, Maluku, Indonesia | 2017 | Madduppa et al., 2015 |
|  | LMBK | 12 | Lombok Island, Indonesia | 2017 | Madduppa et al., 2015 |
|  |  |  |  |  |  |
| Sulawesi Sea |  |  |  |  |  |
|  | BP | 9 | Balikpapan, East Kalimantan, Indonesia | 2017 | Madduppa et al., 2015 |
|  |  |  |  |  |  |
| Northeast Sulawesi |  |  |  |  |  |
|  | D | 3 | Donggi, Central Sulawesi, Indonesia | 2017 | Madduppa et al., 2015 |
|  |  |  |  |  |  |
| Papua |  |  |  |  |  |
|  | MKW | 6 | Manokwari, Papua, Indonesia | 2017 | Madduppa et al., 2015 |
|  |  |  |  |  |  |
| Mariana Islands |  |  |  |  |  |
|  | AI | 14 | Anae island, Guam, USA | 2011 | This study |
|  | DR | 10 | Double reef, Guam, USA | 2011 | This study |
|  |  |  |  |  |  |
| Maldives |  |  |  |  |  |
|  | MDV | 25 | Faafu atoll, Maldives | 2014 | Montano et al., 2014 |
|  |  |  |  |  |  |
| Torres Strait Northern GBR | |  |  |  |  |
|  | GBR | 2 | Lizard island, Great Barrier Reef, Australia | 2010 | Fujii et al., 2011 |

**Supplementary Table 3**: Genetic diversity index from mitochondrial COI sequences with sample number (n), number of haplotypes (*h*), nucleotide diversity(𝝅), haplotype diversity (*h_d_*), p-distance, and standard deviation( SD)

|  | **n** | ***h*** | **π ± SD** | ***h_d_* ± SD** | **p-dist ± SD** |
| --- | --- | --- | --- | --- | --- |
| South China Sea Oceanic Island | 4 | 2 | 0.00085 (0.00081) | 0.50 (0.265) | 0.50 (0.473) |
| South Kuroshio | 28 | 2 | 0.00199 (0.00109) | 0.39 (0.084) | 1.17 (0.632) |
| Mariana Island | 2 | 2 | 0.00511 (0.00279) | 1.00 (0.500) | 3.00 (1.612) |
| Maldives | 5 | 2 | 0.00068 (0.00065) | 0.40 (0.237) | 0.40 (0.373) |
| Northwest Australian Shelf^1^ | 5 | 1 | 0 | 0 | 0 |
| Sulawesi Sea^2^ | 2 | 2 | 0.00511 (0.00279) | 1.00 (0.500) | 3.00 (1.626) |
| Torres Strait Northern GBR | 1 | 1 | - | - | - |
|  | 47 | 5 | 0.00185 (0.00095) | 0.416 (0.00596) | 1.09 (0.547) |
| ^1^ Fromont et al. (2019),  ^2^ Van Der Ent et al. (2015) | | |  |  |  |

**Supplementary Table 4**: Pairwise FST values of *T.hoshinota* in the samples across populations sampled from different regions in the Indo-Pacific.

|  |  | South Kuroshio | South China Sea Ocean Islands | Sunda Shelf | Southern Java | Lesser Sunda | Sulawesi Sea | Northeast Sulawesi | Papua | Mariana Island | Maldives | Torres Strait Northern GBR |
| --- | --- | --- | --- | --- | --- | --- | --- | --- | --- | --- | --- | --- |
| 1 | South Kuroshio | 0 |  |  |  |  |  |  |  |  |  |  |
| 2 | South China Sea Ocean Islands | **0.3641*** | 0 |  |  |  |  |  |  |  |  |  |
| 3 | Sunda Shelf | 0.1 | 0.2 | 0 |  |  |  |  |  |  |  |  |
| 4 | Southern Java | **0.2048*** | 0.1 | 0.1 | 0 |  |  |  |  |  |  |  |
| 5 | Lesser Sunda | 0.1 | **0.2354*** | -0.1 | 0.1 | 0 |  |  |  |  |  |  |
| 6 | Sulawesi Sea | -0 | **0.2914*** | 0 | 0.1 | 0 | 0 |  |  |  |  |  |
| 7 | Northeast Sulawesi | 0.3 | 0 | 0.1 | -0.1 | 0.1 | 0.2 | 0 |  |  |  |  |
| 8 | Papua | **0.3442*** | 0.2 | 0.2 | 0.1 | 0.2 | 0.3 | 0.1 | 0 |  |  |  |
| 9 | Mariana Island | **0.3724*** | 0.1 | 0.2 | 0.1 | **0.2958*** | **0.3327*** | 0.2 | **0.2838*** | 0 |  |  |
| 10 | Maldives | **0.6327*** | **0.5184*** | **0.6687*** | **0.3425*** | **0.6564*** | **0.7431*** | 0.5 | **0.6342*** | **0.5692*** | 0 |  |
| 11 | Torres Strait Northern GBR | **0.6100*** | 0.3 | 0.5 | 0.2 | 0.5 | 0.7 | 0.1 | 0.4 | 0.5 | -0.3 | 0 |

* p<0.001

**Figure S1**: Mismatch distribution graphs for each of the *T.hoshinota* populations. The x axis shows the number of pairwise differences, the y axis shows the frequency of the pairwise comparisons. The red dotted line represented observed frequencies, and the green line represented the frequency expected under the hypothesis of population expansion model.


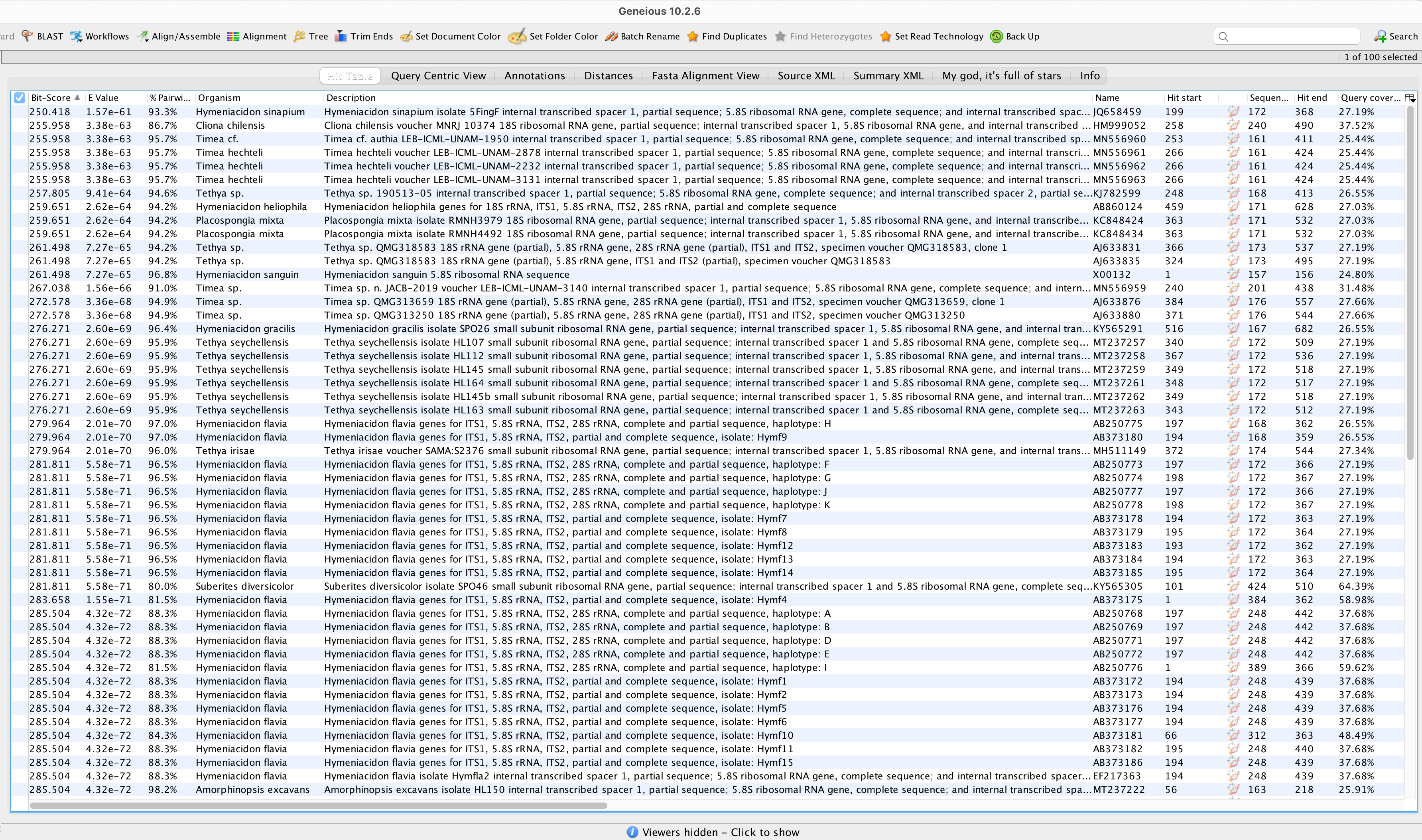


Figure S2:
